# 4-(2-(1*H*-Benzo[*d*]imidazol-2-ylthio)acetamido)-*N*-(substituted phenyl)benzamides: design, synthesis and biological evaluation

**DOI:** 10.1186/s13065-019-0533-7

**Published:** 2019-02-02

**Authors:** Sumit Tahlan, Kalavathy Ramasamy, Siong Meng Lim, Syed Adnan Ali Shah, Vasudevan Mani, Balasubramanian Narasimhan

**Affiliations:** 1 Faculty of Pharmaceutical Sciences, Maharshi Dayanand University, Rohtak, 124001 India; 2 Faculty of Pharmacy, Universiti Teknologi MARA (UiTM), 42300 Bandar Puncak Alam, Selangor Darul Ehsan Malaysia; 3 Collaborative Drug Discovery Research (CDDR) Group, Pharmaceutical Life Sciences Community of Research, Universiti Teknologi MARA (UiTM), 40450 Shah Alam, Selangor Darul Ehsan Malaysia; 4 Atta-ur-Rahman Institute for Natural Products Discovery (AuRIns), Universiti Teknologi MARA (UiTM), Puncak Alam Campus, 42300 Bandar Puncak Alam, Selangor Darul Ehsan Malaysia; 5 Department of Pharmacology and Toxicology, College of Pharmacy, Qassim University, Buraidah, 51452 Kingdom of Saudi Arabia

**Keywords:** *p*-Amino benzoic acid, Antibacterial, Anticancer, Antifungal, 2-Mercaptobenzimidazole derivatives

## Abstract

**Background:**

Dihydrofolate reductase (DHFR) is an important target for antimetabolite class of antimicrobials because it participates in purine synthesis. 2-mercaptobenzimidazole (2MBI) has similar structural features as purine nucleotides. Given that benzimidazole and similar heteroaromatics have been broadly examined for their anticancer potential, so, we hereby report the design, synthesis and biological studies (i.e. antimicrobial and anticancer studies) of 2MBI derivatives.

**Methodology:**

The antimicrobial activity of synthesized 2MBI derivatives were evaluated against Gram positive and Gram negative bacterial species as well as fungal species by tube dilution technique whereas their anticancer activity was assessed against human colorectal carcinoma cell line (HCT116) by Sulforhodamine B (SRB) assay. They were also structurally characterized by IR, NMR, MS and elemental analyses.

**Results, discussion and conclusion:**

The antimicrobial activity findings revealed that compound **N1** (MIC_*bs,st,ca*_ = 1.27, 2.54, 1.27 µM), **N8** (MIC_*ec*_= 1.43 µM), **N22** (MIC_*kp,an*_= 2.60 µM), **N23** and **N25** (MIC_*sa*_= 2.65 µM) exhibited significant antimicrobial effects against tested strains, i.e. Gram-positive, Gram-negative (bacterial) and fungal strains. The anticancer screening results demonstrated that compounds **N9**, **N18** (IC_50_ = 5.85, 4.53 µM) were the most potent compounds against cancer cell line (HCT116) even more than 5-FU, the standard drug (IC_50_ = 9.99 µM). 
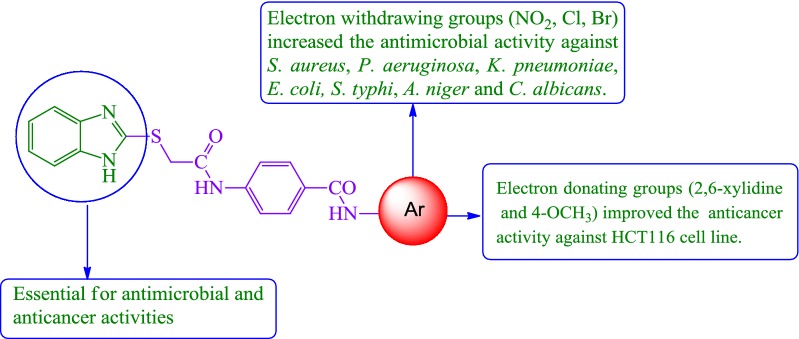

## Background

The global statistics of infectious diseases have reached a worrying level following the rise of multi-drug resistant Gram-positive and Gram-negative microorganisms. Advanced treatments against infections which rely on a long multidrug schedule are often impeded by patient noncompliance and the emergence of multidrug resistant pathogens. The issue of resistance could be potentially overcome by exploring new effective agents. In this regard, rational drug design has been proven to be much beneficial as the biochemical basis of mechanisms of intrinsic and acquired resistance is largely known [1].

In terms of cancer, presently used chemotherapeutic agents restrain the growth of tumour through suppression of DNA replication and transcription. Chemotherapy is, however, often compromised by development of multidrug resistance, endurance of cancer cells in anaerobic environment, involvement of ribonucleotide reductase (RNR), topoisomerase I (Topo I) and topoisomerase II (Topo II) in neoplasm growth. Nevertheless, the attempt of discovering new curative anticancer agents in last decade has led to targets of specific molecular modifications in tumour cells. The new approach now focuses mainly on the development of small biologically active molecules containing significant activity without toxicity related to the usual chemotherapy [2].

Substituted benzimidazole compounds, with excellent antimicrobial activity against bacteria and ability to permeate mammalian cell membranes as well as maintain the activity with low to no associated toxicity have made these lead compounds attractive for further research work in the field of medicine [3]. The search for new active, antitumour drugs with lower toxicity against normal cells and tissues, however, remains as one of the most significant problems of modern antitumour chemotherapy [4].

**Fig. 1:**
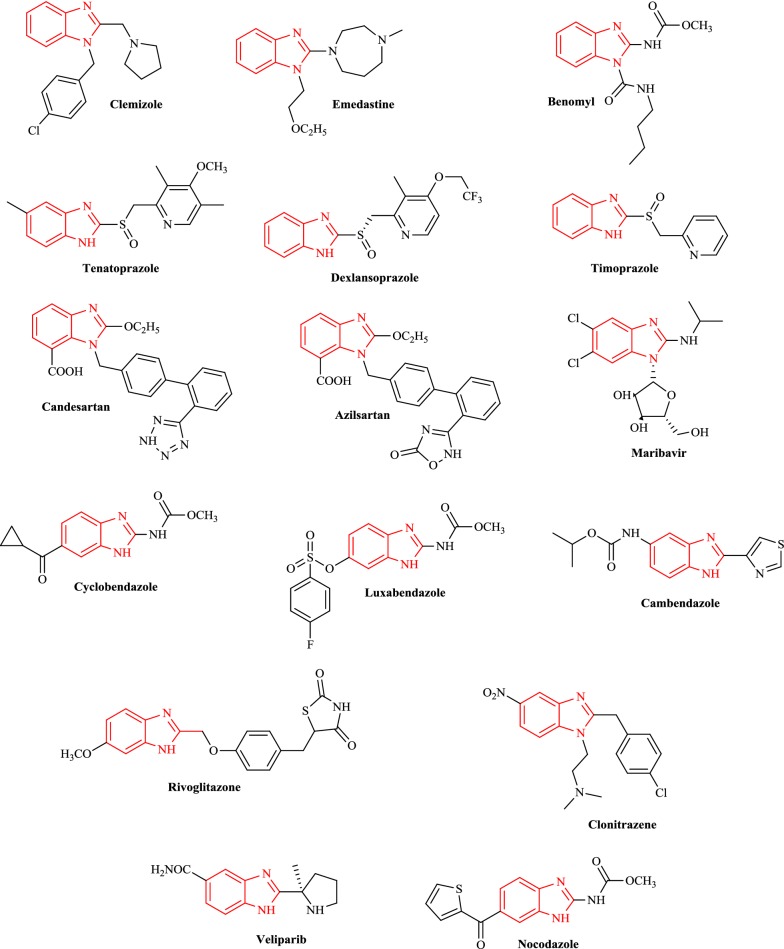
Marketed formulations having benzimidazole moiety

In modern drug discovery and medicinal chemistry, benzimidazole nucleus has been proven to be a very significant pharmacophore with a wide variety of activities [5] including antihistaminic (H_1_-receptor antagonists, e.g. clemizole and emedastine) [6], antifungal (systemic fungicide, e.g. benomyl) [7], antiulcer (proton pump inhibitors (PPIs), e.g. tenatoprazole, dexlansoprazole and timoprazole) [8], antihypertensive (angiotensin II receptor blockers, e.g. candesartan and azilsartan) [9], antiviral (oral ribosyl benzimidazole, e.g. maribavir) [10], antiparasitic (specifically anthelmintics, e.g. cyclobendazole, luxabendazole and cambendazole) [11], antidiabetic (PPAR gamma agonist, e.g. rivoglitazone) [12], analgesic (opioid analgesic, e.g. clonitrazene) [13] and anticancer (antimitotic agent, e.g. nocodazole, PARP inhibitor, e.g. veliparib) [14] (Fig. 1).

**Fig. 2:**
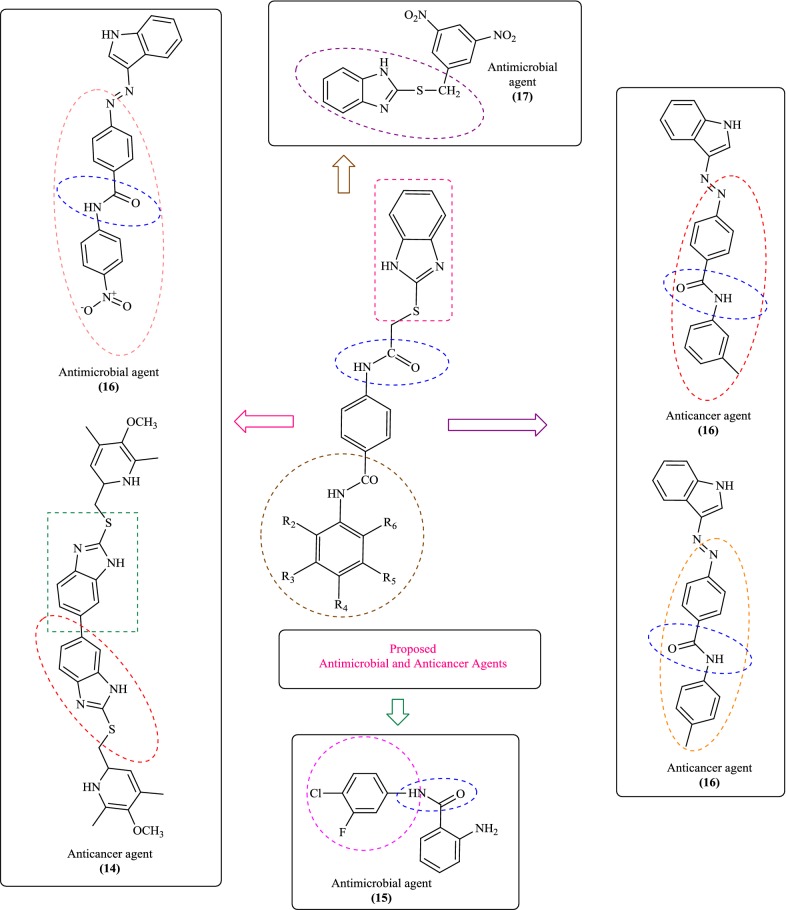
Design of benzimidazole analogues for antimicrobial and anticancer activity based on literature study

Figure 2 shows some antimicrobial and anticancer activities of benzimidazole derivatives which were reviewed based on a literature survey [15–19]. As part of our continuing effort in search for new therapeutic agents, we had synthesized a number of pharmacologically important heterocyclic compounds (**N1–N26**). In order to study the impact of electron-donating and accepting groups within the moieties, an attempt has been made to combine 2MBI and *p*-amino benzoic acid moieties as hybrid and substitute them with different substituted amines. In this regard, a library of substituted benzimidazole was designed, synthesized, spectroscopically characterized and evaluated for potential antimicrobial and anticancer effects in vitro.

## Results and discussion

### Chemistry

**Scheme 1:**
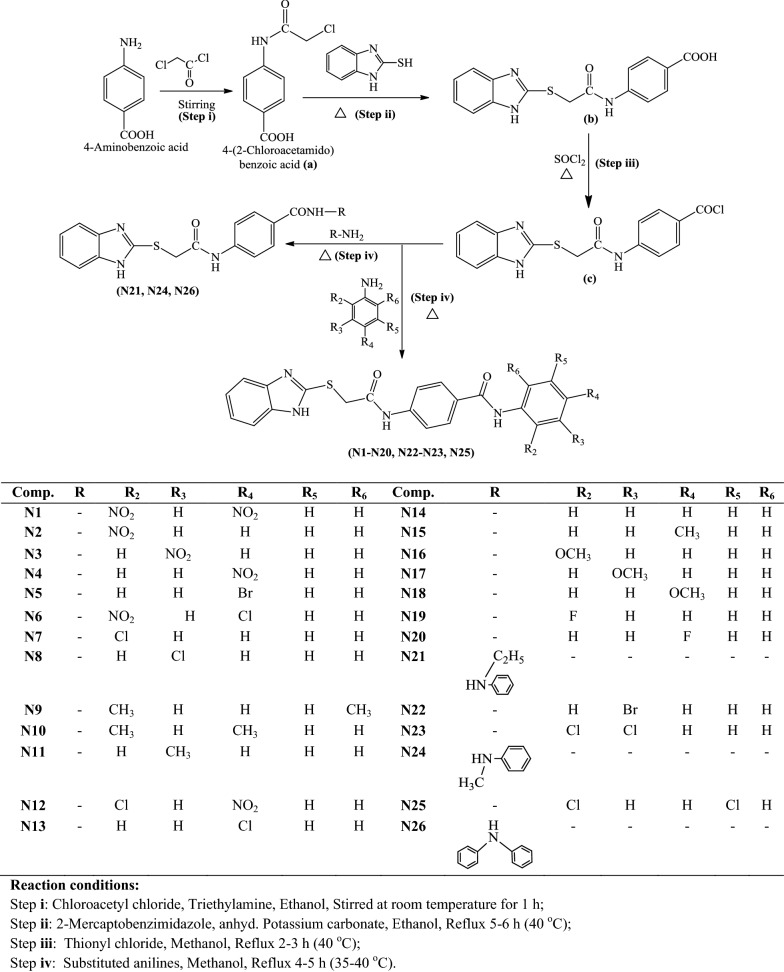
Synthesis of 4-(2-(1*H*-benzo[*d*]imidazol-2-ylthio)acetamido)-*N*-(substitutedphenyl) benzamide derivatives

A series of 2MBI benzamides has been synthesized as discussed in Scheme 1. The 4-(2-chloro acetamido) benzoic acid **(a)** was prepared by the reaction of chloroacetyl chloride with *p*-amino benzoic acid, which on further reaction with 2-mercaptobenzimidazole yielded 4-(2-(1*H*-benzo[*d*]imidazol-2-ylthio)acetamido) benzoic acid **(b)**. The reaction of **b** with thionyl chloride resulted in the formation of 4-(2-(1*H*-benzo[*d*]imidazol-2-ylthio)acetamido)benzoyl chloride **(c)**. The reaction of above synthesized benzoyl chloride **(c)** with different substituted anilines in methanolic/ethanolic solvent yielded the title scaffolds (**N1**–**N26**). The physicochemical properties, elemental analyses and mass spectral studies of title compounds (**N1**–**N26**) are presented in Table 1. The molecular structures of synthesized compounds were determined by FT-IR, ^1^H/^13^C-NMR (Table 2). The halogenated compounds, **N5** and **N22** (Ar–Br) showed peak around 633–616 cm^−1^, **N19** and **N20** (Ar-F) at 1117–1116 cm^−1^ and **N6**, **N7**, **N8**, **N12**, **N13**, **N23** and **N25 (**Ar–Cl) around 771–738 cm^−1^. The existence of Ar–NO_2_ group (**N1**, **N2**, **N3**, **N4**, **N6** and **N12)** was indicated by the appearance of stretching at 1549–1513 cm^−1^. The spectral data of aryl alkyl ether group (C–O–C, Ar–OCH_3_) in compounds **N16**, **N17** and **N18** was in between 2837 and 2831 cm^−1^. The band at 2948–2860 cm^−1^ in the spectral data of **N9**, **N10**, **N11** and **N15** depicted the presence of Ar–CH_3_. The IR stretching around 714–689 cm^−1^ (C–S), 1692–1635 cm^−1^ (–CONH–), 3110–3018 cm^−1^, ~ 1600 cm^−1^ (C–H and C=C, Ar) and 1361–1253 cm^−1^ (C=N) found in all synthesized 2MBI derivatives. In ^1^H-NMR spectra, the existence of multiplet signals between 6.51 and 9.10 δ ppm indicated the presence of aromatic proton of synthesized derivatives (**N1**–**N26**). Due to presence of Ar-CH_3_, compounds **N9**, **N10**, **N11** and **N15** showed singlet at 2.23–2.53 δ ppm and because of Ar–OCH_3_, **N16**, **N17** and **N18** showed singlet at range of 3.73–3.74 δ ppm. The synthesized molecules exhibited singlet at 7.93–8.01 δ ppm, 2.55–4.38 δ ppm and 4.36–4.53 δ ppm due to the existence of –CONH, –CH_2_ groups and –NH of imidazole ring. The findings of elemental analyses of synthesized 2MBI derivatives were recorded within theoretical results of ± 0.4%. Conclusively, the ^13^C-NMR spectra of synthesized benzamides were in DMSO-*d6* and their molecular structures were in accordance with the spectral signals. Mass spectra of the synthesized derivatives reflected the characteristic molecular ion peaks.

**Table 1:**
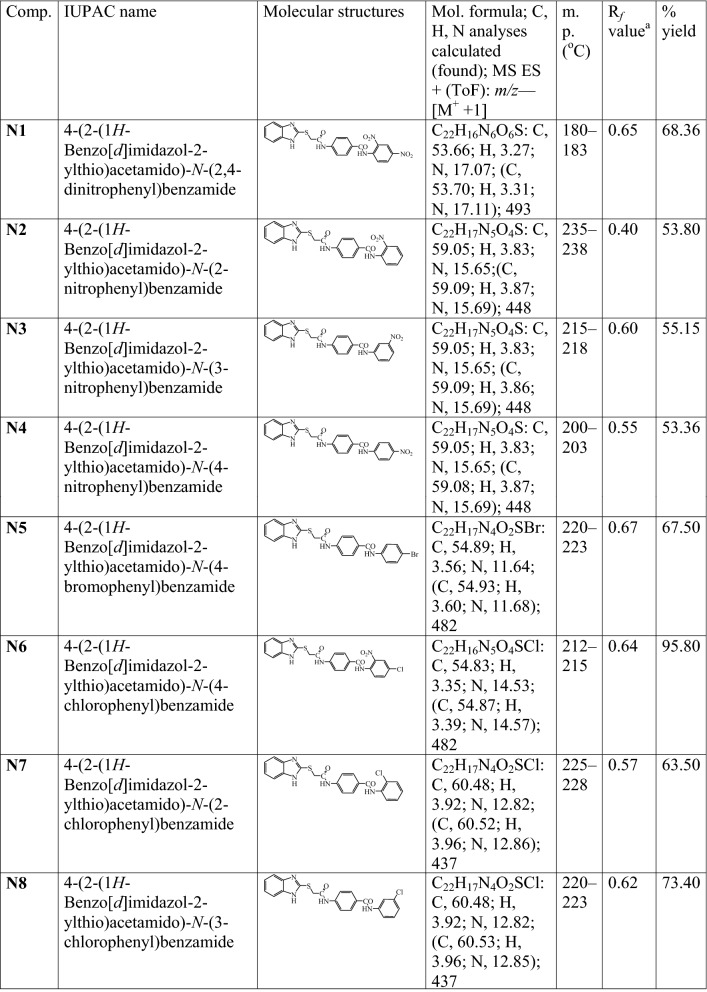

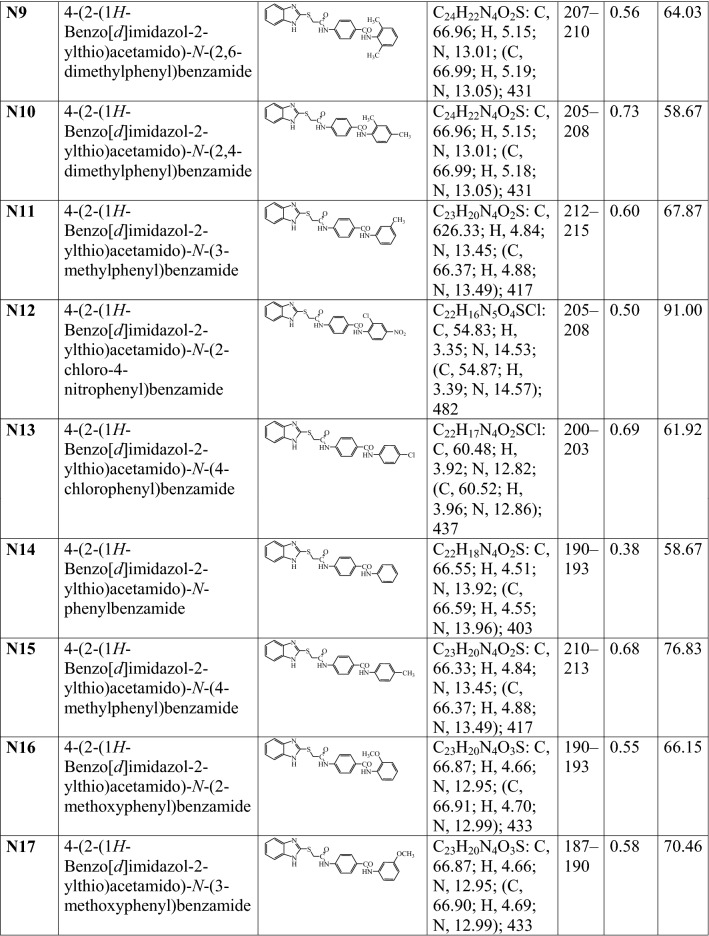

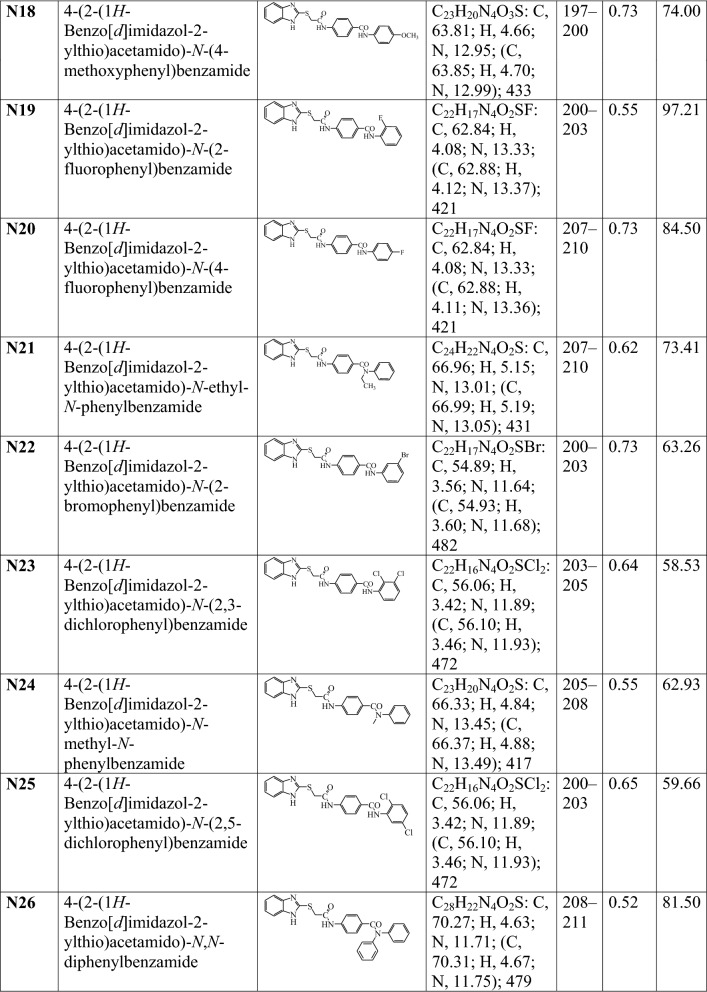
The physicochemical properties of synthesized 2-mercaptobenzimidazole derivatives

^**a**^TLC mobile phase-Ethyl acetate

**Table 2:** Spectral interpretation of synthesized derivatives

Comp.	IR (KBr cm^−1^)	^13^C-NMR (DMSO-*d6*, δ ppm)	^1^H-NMR (DMSO-*d6*, δ ppm)
**N1**	[3104 (C–H str.), 1602 (C=C str.) pn(phenyl nucleus)], 1626 (–CONH str.), 1333 (N=CH str. (3^0^ amine)), 1256 (C–N str. (2^0^ amine)), 714 (C–S str., CH_2_–S), 2838 (C–H str., –CH_2_–), 1516 (C–NO_2_ str., C_6_H_5_NO_2_)	39.02, 113.68, 118.35, 122.48, 123.21, 128.54, 129.10, 137.44, 137.45, 142.67, 149.72, 166.25, 166.78	7.25–8.77 (m, 11H, Ar–H), 4.42 (s, 1H, NH of imidazole), 7.93 (s, 2H, (CONH)_2_)
**N2**	[3058 (C–H str.), 1599 (C=C str.) pn, 1684 (–CONH str.), 1332 (N=CH str.), 1293 (C–N str.), 716 (C–S str., CH_2_–S), 2931 (C–H str., –CH_2_–), 1513 (C–NO_2_ str., C_6_H_5_NO_2_)	3.6.41, 115.43, 118.44, 119.13, 122.59, 125.47, 130.25, 130.43, 135.62, 137.42, 142.72, 149.80, 166.28, 166.83	7.25–7.66 (m, 12H, Ar–H), 4.45 (s, 1H, NH of imidazole), 7.96 (s, 2H, (CONH)_2_)
**N3**	[3109 (C–H str.), 1599 (C=C str.) pn], 1666 (–CONH str.), 1331 (N=CH str.), 1299 (C–N str.), 713 (C–S str., CH_2_–S), 2833 (C–H str., –CH_2_–), 1540 (C–NO_2_ str., C_6_H_5_NO_2_)	36.42, 113.65, 118.42, 120.05, 122.48, 125.46, 129.83, 130.43, 137.43, 137.63, 142.74, 149.80, 166.32, 166.83	7.24–7.97 (m, 12H, Ar–H), 4.45 (s, 1H, NH of imidazole), 7.97 (s, 2H, (CONH)_2_)
**N4**	[3110 (C–H str.), 1600 (C=C str.) pn], 1681 (–CONH str.), 1301 (N=CH str.), 1280 (C–N str.), 712 (C–S str., CH_2_–S), 2934 (C–H str., –CH_2_–), 1542 (C–NO_2_ str., C_6_H_5_NO_2_)	113.63, 118.43, 118.55, 122.62, 125.47, 126.31, 130.43, 137.34, 142.73, 149.81, 166.27, 166.82;	7.26–7.97 (m, 12H, Ar–H), 4.47 (s, 1H, NH of imidazole), 7.97 (s, 2H, (CONH)_2_)
**N5**	3107 (C–H str.), 1598 (C=C str.)pn], 1669 (–CONH str.), 1330 (N=CH str.), 1313 (C–N str.), 711 (C–S str., CH_2_–S), 2944 (C–H str., –CH_2_–), 633 (C–Br str., C_6_H_5_Br)	39.02, 113.79, 118.38, 121.81, 125.42, 130.79, 149.72, 166.58, 166.84	7.19–7.97 (m, 12H, Ar–H), 4.38 (s, 1H, NH of imidazole), 4.34 (s, 1H, CH_2_), 7.97 (s, 2H, (CONH)_2_)
**N6**	[3101 (C–H str.), 1599 (C=C str.) pn], 1635 (–CONH str.), 1338 (N=CH str.), 1293 (C–N str.), 693 (C–S str., CH_2_–S), 2833 (C–H str., –CH_2_–),762 (C–Cl str., C_6_H_5_Cl), 1549 (C–NO_2_ str., C_6_H_5_NO_2_)	36.47, 113.56, 121.13, 122.84, 123.98, 125.41, 125.47, 129.95, 130.41, 130.43, 135.31, 136.81, 142.65, 142.70, 149.84, 166.15, 166.80	7.27–7.95 (m, 11H, Ar–H), 4.47 (s, 1H, NH of imidazole), 7.95 (s, 2H, (CONH)_2_)
**N7**	[3109 (C–H str.), 1599 (C=C str.) pn], 1686 (–CONH str.), 1318 (N=CH str.), 1293 (C–N str.), 694 (C–S str., CH_2_–S), 2870 (C–H str., –CH_2_–),750 (C–Cl str., C_6_H_5_Cl)	36.41, 115.59, 118.41, 122.56, 125.45, 125.56, 127.60, 128.92, 130.43, 137.29, 137.49, 142.73, 149.80, 166.27, 166.80	7.23–7.97 (m, 12H, Ar–H), 4.42 (s, 1H, NH of imidazole), 7.93 (s, 2H, (CONH)_2_)
**N8**	[3106 (C–H str.), 1598 (C=C str.) pn, 1683 (–CONH str.), 1331 (N=CH str.), 1293 (C–N str.), 694 (C–S str., CH_2_–S), 2868 (C–H str., –CH_2_–), 750 (C–Cl str., C_6_H_5_Cl)	36.38, 113.67, 118.44, 122.42, 125.46, 130.44, 135.62, 137.68, 142.73, 149.76, 166.38, 166.86;	7.19–7.94 (m, 12H, Ar–H), 4.41 (s, 1H, NH of imidazole), 7.94 (s, 2H, (CONH)_2_)
**N9**	[3018 (C–H str.), 1598 (C=C str.) pn], 1668 (–CONH str.), 1360 (N=CH str.), 1281 (C–N str.), 713 (C–S str., CH_2_–S), 2915 (C–H str., –CH_2_–), 2948 (C–H str., CH_3_)	39.01, 118.37, 121.64, 125.41, 130.46, 142.81, 149.71, 166.65, 166.85;	7.16–7.96 (m, 11H, Ar–H), 4.36 (s, 1H, NH of imidazole), 7.97 (s, 2H, (CONH)_2_), 2.53 (s, 6H, (–CH_3_)_2_)
**N10**	[3055 (C–H str.), 1599 (C=C str.)pn], 1667 (–CONH str.), 1331 (N=CH str.), 1298 (C–N str.), 714 (C–S str., CH_2_–S), 2934 (C–H str., –CH_2_–), 2888 (C–H str., CH_3_)	17.63, 20.37, 38.99, 113.78, 118.39, 121.82, 124.03, 125.42, 126.43, 130.46, 130.76, 138.78, 138.84, 142.79, 149.72, 166.59, 166.85	7.18–7.96 (m, 11H, Ar–H), 4.39 (s, 1H, NH of imidazole), 4.30 (s, 2H, CH_2_), 7.98 (s, 2H, (CONH)_2_), 2.23 (s, 6H, (–CH_3_)_2_)
**N11**	[3054 (C–H str.), 1597 (C=C str.) pn], 1667 (–CONH str.), 1360 (N=CH str.), 1311 (C–N str.), 713 (C–S str., CH_2_–S), 2834 (C–H str., –CH_2_–), 2877 (C–H str., CH_3_)	21.12, 39.01, 116.25, 118.45, 121.73, 124.18, 125.41, 128.59, 130.39, 130.46, 137.99, 138.72, 138.80, 138.92, 142.80, 149.73, 166.62, 166.85	7.17–7.96 (m, 12H, Ar–H), 4.39 (s, 1H, NH of imidazole), 4.33 (s, 2H, CH_2_), 7.97 (s, 2H, (CONH)_2_), 2.28 (s, 3H, –CH_3_)
**N12**	[3047 (C–H str.), 1604 (C=C str.) pn], 1650 (–CONH str.), 1318 (N=CH str.), 1253 (C–N str.), 692 (C–S str., CH_2_–S), 2905 (C–H str., –CH_2_–), 744 (C–Cl str., C_6_H_5_Cl), 1546 (C–NO_2_ str., C_6_H_5_NO_2_)	36.54, 115.49, 118.44, 123.23, 124.53, 125.50, 125.55, 130.40, 135.87, 142.68, 149.88, 166.02, 166.81	7.31–8.11 (m, 11H, Ar–H), 4.53 (s, 1H, NH of imidazole), 2.55 (s, 2H, CH_2_), 7.98 (s, 2H, (CONH)_2_)
**N13**	3051 (C–H str.), 1598 (C=C str.) pn], 1631 (–CONH str.), 1331 (N=CH str.), 1315 (C–N str.), 694 (C–S str., CH_2_–S), 2951 (C–H str., –CH_2_–), 749 (C–Cl str., C_6_H_5_Cl)	38.96, 113.67, 118.42, 122.39, 125.45, 130.44, 137.72, 142.74, 149.77, 166.37, 166.84	7.31–7.94 (m, 12H, Ar–H), 4.53 (s, 1H, NH of imidazole), 7.97 (s, 2H, (CONH)_2_)
**N14**	[3059 (C–H str.), 1599 (C=C str.) pn, 1683 (–CONH str.), 1333 (N=CH str.), 1315 (C–N str.), 692 (C–S str., CH_2_–S), 2921 (C–H str., –CH_2_–)	39.02, 113.67, 118.40, 122.31, 122.38, 123.52, 125.43, 128.77, 130.43, 137.85, 138.74, 142.75, 149.77, 166.79, 166.82;	7.07–7.93 (m, 13H, Ar–H), 4.41 (s, 1H, NH of imidazole), 4.38 (s, 2H, CH_2_), 7.94 (s, 2H, (CONH)_2_)
**N15**	[3053 (C–H str.), 1599 (C=C str.) pn], 1667 (–CONH str.), 1361 (N=CH str.), 1310 (C–N str.), 714 (C–S str., CH_2_–S), 2934 (C–H str., –CH_2_–), 2860 (C–H str., CH_3_)	36.26, 118.34, 119.02, 121.52, 125.37, 129.14, 130.45, 132.39, 136.32, 142.81, 149.68, 165.83, 166.82	7.11–7.92 (m, 12H, Ar–H), 4.34 (s, 1H, NH of imidazole), 4.28 (s, 2H, CH_2_), 7.94 (s, 2H, (CONH)_2_), 2.51 (s, 3H, –CH_3_)
**N16**	[3055 (C–H str.), 1599 (C=C str.) pn], 1683 (–CONH str.), 1332 (N=CH str.), 1315 (C–N str.), 694 (C–S str., CH_2_–S), 2941 (C–H str., –CH_2_–), 1250 (C–O–C str., phenyl ether), 2837 (C–H str., O-CH_3_)	38.87, 113.64, 118.46, 122.51, 125.48, 130.44, 137.50, 142.72, 149.77, 166.36, 166.88	7.26–7.82 (m, 12H, Ar–H), 4.49 (s, 1H, NH of imidazole), 4.38 (s, 2H, CH_2_), 8.00 (s, 2H, (CONH)_2_), 3.73 (s, 3H, –OCH_3_)
**N17**	[3107 (C–H str.), 1599 (C=C str.) pn], 1685 (–CONH str.), 1332 (N=CH str.), 1316 (C–N str.), 694 (C–S str., CH_2_–S), 2917 (C–H str., –CH_2_–), 1250 (C–O–C str., phenyl ether), 2837 (C–H str., O-CH_3_)	38.93, 113.67, 118.43, 122.38, 125.45, 130.44, 137.75, 142.74, 149.77, 166.39, 166.86	7.24–7.98 (m, 12H, Ar–H), 4.45 (s, 1H, NH of imidazole), 4.40 (s, 2H, CH_2_), 7.98 (s, 2H, (CONH)_2_), 3.74 (s, 3H, –OCH_3_)
**N18**	[3054 (C–H str.), 1600 (C=C str.) pn], 1679 (–CONH str.), 1334 (N=CH str.), 1314 (C–N str.), 691 (C–S str., CH_2_–S), 2933 (C–H str., –CH_2_–), 1248 (C–O–C str., phenyl ether), 2831 (C–H str., O-CH_3_)	38.95, 55.08, 113.89, 118.40, 120.66, 121.67, 125.43, 130.48, 131.92, 139.06, 149.72, 155.38, 165.61, 166.88;	6.92–7.99 (m, 12H, Ar–H), 4.40 (s, 1H, NH of imidazole), 4.33 (s, 2H, CH_2_), 7.99 (s, 2H, N(CONH)_2_), 3.74 (s, 3H, –OCH_3_)
**N19**	[3106 (C–H str.), 1597 (C=C str.) pn], 1667 (–CONH str.), 1361 (N=CH str.), 1313 (C–N str.), 692 (C–S str., CH_2_–S), 2934 (C–H str., –CH_2_–), 1117 (C–F str., C_6_H_5_F)	38.93, 113.64, 118.44, 122.52, 125.47, 130.44, 137.46, 142.74, 149.79, 166.34, 166.86	7.26–7.99 (m, 12H, Ar–H), 4.49 (s, 1H, NH of imidazole), 8.00 (s, 2H, (CONH)_2_)
**N20**	[3066 (C–H str.), 1598 (C=C str.) pn], 1667 (–CONH str.), 1361 (N=CH str.), 1310 (C–N str.), 713 (C–S str., CH_2_–S), 2937 (C–H str., –CH_2_–), 1116 (C–F str., C_6_H_5_F)	39.03, 118.36, 121.63, 125.39, 130.46, 142.80, 149.70, 166.64, 166.83	7.15–7.94 (m, 12H, Ar–H), 4.36 (s, 1H, NH of imidazole), 4.31 (s, 2H, CH_2_), 7.95 (s, 1H, CONH)
**N21**	[3103 (C–H str.), 1597 (C=C str.) pn], 1666 (–CONH str.), 1360 (N=CH str.), 1310 (C–N str.), 711 (C–S str., CH_2_–S), 2935 (C–H str., –CH_2_–), 2911 (C–H str., CH_3_)	39.03, 118.36, 121.63, 125.40, 130.46, 142.80, 149.70, 166.64, 166.83	7.16–7.96 (m, 13H, Ar–H), 4.36 (s, 1H, NH of imidazole), 7.96 (s, 1H, CONH)
**N22**	[3104 (C–H str.), 1599 (C=C str.) pn], 1684 (–CONH str.), 1331 (N=CH str.), 1316 (C–N str.), 695 (C–S str., CH_2_–S), 28,668 (C–H str., –CH_2_–), 616 (C–Br str., C_6_H_5_Br)	39.03, 113.68, 118.40, 122.36, 125.43, 130.43, 137.78, 142.75, 149.78, 166.36, 166.81	7.23–7.94 (m, 12H, Ar–H), 4.41 (s, 1H, NH of imidazole), 7.94 (s, 2H, (CONH)_2_)
**N23**	[3107 (C–H str.), 1602 (C=C str.) pn], 1650 (–CONH str.), 1350 (N=CH str.), 1316 (C–N str.), 691 (C–S str., CH_2_–S), 2840 (C–H str., –CH_2_–), 738 (C–Cl str., C_6_H_5_Cl)	39.02, 114.73, 118.53, 123.26, 125.50, 127.92, 130.41, 131.50, 136.04, 146.61, 166.02, 166.81	6.80–7.94 (m, 11H, Ar–H), 4.53 (s, 1H, NH of imidazole), 7.95 (s, 2H, (CONH)_2_)
**N24**	[3108 (C–H str.), 1599 (C=C str.) pn], 1685 (–CONH str.), 1332 (N=CH str.), 1316 (C–N str.), 694 (C–S str., CH_2_–S), 2916 (C–H str., –CH_2_–), 2874 (C–H str., CH_3_)	38.98, 113.61, 118.43, 122.64, 125.46, 130.42, 137.23, 142.72, 149.80, 166.27, 166.83	7.26–7.95 (m, 13H, Ar–H), 4.46 (s, 1H, NH of imidazole), 7.96 (s, 1H, CONH)
**N25**	[3109 (C–H str.), 1604 (C=C str.) pn], 1651 (–CONH str.), 1351 (N=CH str.), 1318 (C–N str.), 691 (C–S str., CH_2_–S), 2837 (C–H str., –CH_2_–), 771 (C–Cl str., C_6_H_5_Cl)	36.52, 115.62, 118.45, 123.13, 125.49, 130.18, 130.42, 131.91, 136.21, 136.27, 142.70, 149.87, 166.07, 166.82;	^1^H-NMR: 6.88–7.95 (m, 11H, Ar–H), 4.52 (s, 1H, NH of imidazole), 7.96 (s, 2H, (CONH)_2_)
**N26**	[3107 (C–H str.), 1600 (C=C str.) pn, 1650 (–CONH str.), 1351 (N=CH str.), 1316 (C–N str.), 689 (C–S str., CH_2_–S), 2839 (C–H str., –CH_2_–), 1650 (phenyl conjugation)	36.55, 116.67, 118.41, 119.57, 123.21, 125.48, 125.51, 129.07, 130.43, 136.15, 142.72, 143.38, 149.90, 166.05, 166.83	7.10–7.56 (m, 17H, Ar–H), 4.53 (s, 1H, NH of imidazole), 7.96 (s, 1H, CONH)

### Antimicrobial screening results

**Fig. 3:**
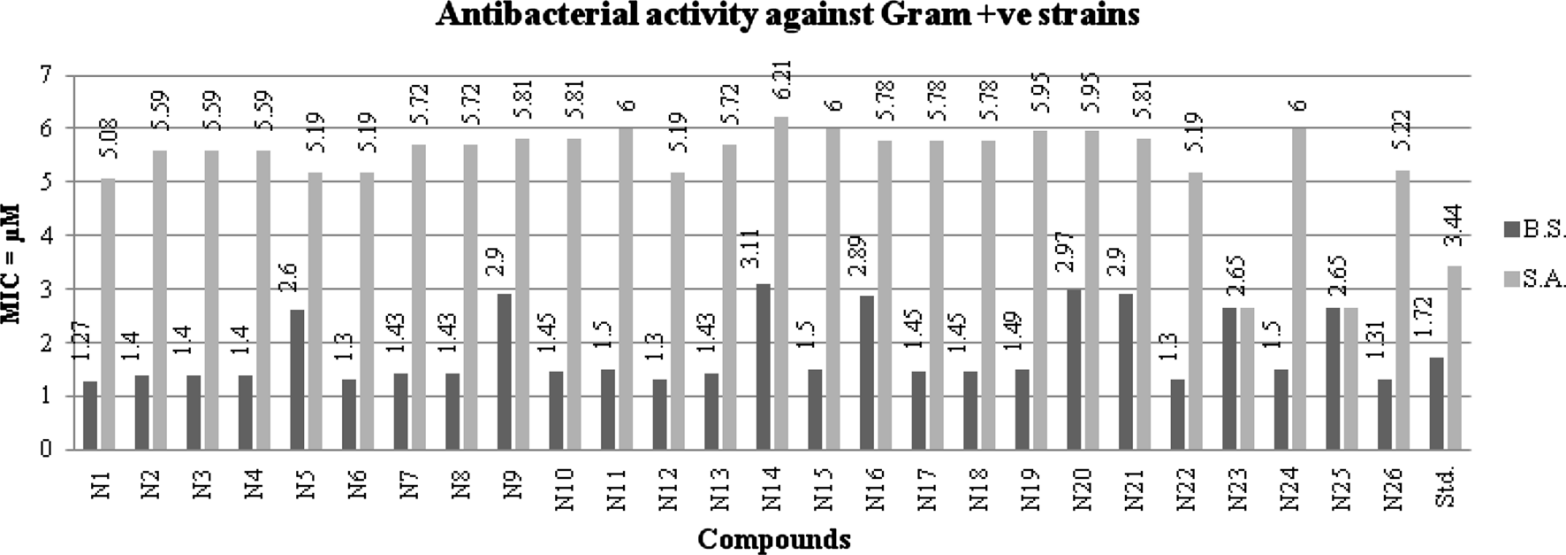
Antibacterial screening results against Gram positive species

**Fig. 4:**
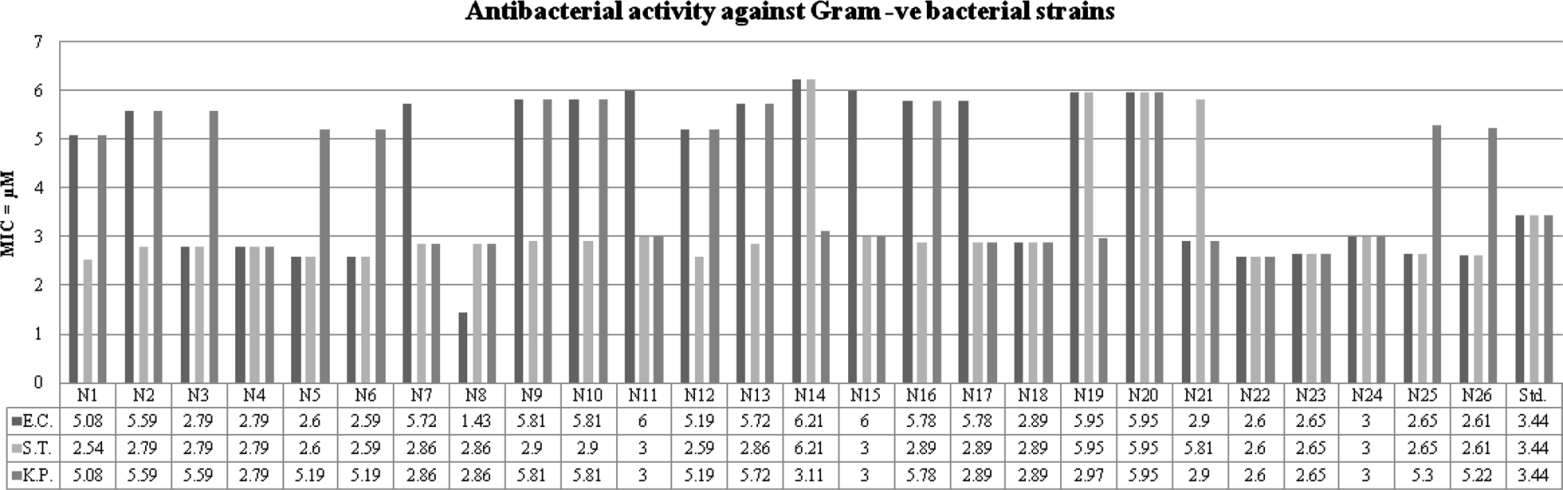
Antibacterial screening results against Gram negative species

**Fig. 5:**
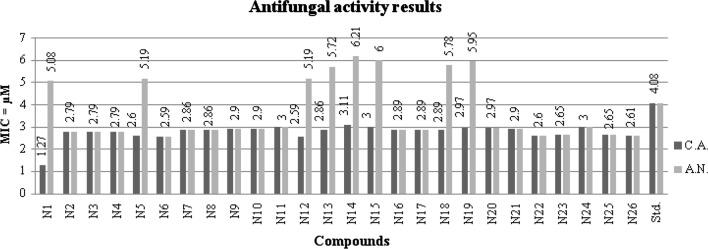
Antifungal screening results against fungal species

The results of synthesized 2MBI benzamides (**N1**–**N26**) are presented in Table 3, Figs. 3, 4 and 5. The synthesized compounds were screened for their antimicrobial potential using cefadroxil (antibacterial) and fluconazole (antifungal) as standard drugs. Among the synthesized compounds, 2, 4-dinitro substituted benzamide, i.e. compound **N1** (MIC_*bs,st,*ca_ = 1.27, 2.54, 1.27 µM) exhibited significantly potent antimicrobial effect against *B. subtilis*, *S. typhi* and *C. albicans*, respectively. The *meta* substituted (–Cl and –Br) derivatives, i.e. compounds **N8** (MIC_*ec*_ = 1.43 µM) and **N22** (MIC_*kp,an*_ = 2.60 µM) displayed promising activity against *E. coli*, *K. pneumoniae* and *A. niger*, respectively. Furthermore, the 2, 3 and 2,5-dichloro substituted ones, i.e. compounds **N23** and **N25** (MIC_*sa*_ = 2.65µM) were found to be more potent against *S. aureus*. From the antimicrobial screening results, it was found that all the scaffolds had excellent activity compared with the reference drugs.

**Table 3:** Antimicrobial and anticancer screening results of synthesized compounds

Compounds	MIC (minimum inhibitory concentration = µM)							IC_50_ (μM)
	Bacterial strains					Fungal strains		Cancer cell line (HCT116)
	Gram +ve		Gram −ve					
	MIC_*bs*_	MIC_*sa*_	MIC_*ec*_	MIC_*st*_	MIC_*kp*_	MIC_*ca*_	MIC_*an*_	
**N1**	1.27	5.08	5.08	2.54	5.08	1.27	5.08	> 20.31
**N2**	1.40	5.59	5.59	2.79	5.59	2.79	2.79	> 22.35
**N3**	1.40	5.59	2.79	2.79	5.59	2.79	2.79	> 22.35
**N4**	1.40	5.59	2.79	2.79	2.79	2.79	2.79	> 22.35
**N5**	2.60	5.19	2.60	2.60	5.19	2.60	5.19	> 20.77
**N6**	1.30	5.19	2.59	2.59	5.19	2.59	2.59	> 20.75
**N7**	1.43	5.72	5.72	2.86	2.86	2.86	2.86	> 22.89
**N8**	1.43	5.72	1.43	2.86	2.86	2.86	2.86	13.73
**N9**	2.90	5.81	5.81	2.90	5.81	2.90	2.90	5.85
**N10**	1.45	5.81	5.81	2.90	5.81	2.90	2.90	> 23.23
**N11**	1.50	6.00	6.00	3.00	3.00	3.00	3.00	> 24.01
**N12**	1.30	5.19	5.19	2.59	5.19	2.59	5.19	> 20.75
**N13**	1.43	5.72	5.72	2.86	5.72	2.86	5.72	> 22.89
**N14**	3.11	6.21	6.21	6.21	3.11	3.11	6.21	12.42
**N15**	1.50	6.00	6.00	3.00	3.00	3.00	6.00	19.21
**N16**	2.89	5.78	5.78	2.89	5.78	2.89	2.89	> 23.12
**N17**	1.45	5.78	5.78	2.89	2.89	2.89	2.89	> 23.12
**N18**	1.45	5.78	2.89	2.89	2.89	2.89	5.78	4.53
**N19**	1.49	5.95	5.95	5.95	2.97	2.97	5.95	> 23.78
**N20**	2.97	5.95	5.95	5.95	5.95	2.97	2.97	14.27
**N21**	2.90	5.81	2.90	5.81	2.90	2.90	2.90	> 23.23
**N22**	1.30	5.19	2.60	2.60	2.60	2.60	2.60	> 13.86
**N23**	2.65	2.65	2.65	2.65	2.65	2.65	2.65	> 21.22
**N24**	1.50	6.00	3.00	3.00	3.00	3.00	3.00	20.41
**N25**	2.65	2.65	2.65	2.65	5.30	2.65	2.65	> 21.22
**N26**	1.31	5.22	2.61	2.61	5.22	2.61	2.61	> 20.90
Broth control	NG	NG	NG	NG	NG	NG	NG	–
Std.	1.72^a^	3.44^a^	3.44^a^	3.44^a^	3.44^a^	4.08^b^	4.08^b^	9.99^c^

*Bacillus subtilis MTCC 441*-*bs, Staphylococcus aureus MTCC 3160*-*sa, Escherichia coli MTCC 443*-*ec, Salmonella typhi MTCC 3231*-*st, Klebsiella pneumoniae MTCC 9024*-*kp, Candida albicans MTCC 227*-ca *and Aspergillus niger MTCC 281*-*an*

*DMSO* dimethyl sulfoxide, *NG* no growth

Std. drugs: ^a^ Cefadroxil; ^b^ Fluconazole; ^c^ 5-FU (5-Fluorouracil)

### Anticancer screening results

The anticancer activity of synthesized analogues (**N1**–**N26**) was evaluated against human colorectal cancer cell line (HCT116) by SRB assay. Table 3 shows the antiproliferative outcome of the synthesized analogues and 5-FU, the standard drug against HCT116. The anticancer screening results indicated that 2,6-xylidine and *para*-methoxy substituted scaffolds, i.e. compounds **N9** (IC_50_ = 5.85 µM) and **N18** (IC_50_ = 4.53 µM) to exhibit the greatest anticancer activity among the synthesized scaffolds. The anticancer effect was even more potent than 5-FU (IC_50_ = 9.99 µM).

### Toxicity study

For the selectivity index calculation of the most active compounds (**N9** and **N18**), these compounds were tested against normal human embryonic kidney cell line (HEK-293). Compounds were dissolved into 0.1% DMSO solution. The compounds were diluted in concentration (2 μM, 4 μM, 6 μM, 8 μM and 10 μM). The cells were incubated with these compounds for 24 h and more than almost 100% of HEK-293 cells were viable at IC_50_ for growth inhibition of each studied compound. Results showed the significant viability difference between the test compounds treated and control cells (at zero concentration) after 24 h with (P < 0.01). The 50% of the cells were viable at the lethal dose (LD_50_) 8.43 μM and 8.22 μM of the active compounds, **N9** and **N18**, respectively. As we know that higher the LD_50_ value than the IC_50_ higher will be the selectivity that implied that the compounds may have better safety of the each of two compounds since the IC_50_ is much lower the LD_50_ (Table 4).

**Table 4:** Lethal dose and selectivity index calculation of most active compounds (**N9** and **N18**)

Compound	Lethal dose (LD_50_)	IC_50_	Selectivity index (LD_50_/IC_50_)
**N9**	8.43	5.85	1.44
**N18**	8.22	4.53	1.81

### MTT assay

Human embryonic kidney (HEK-293) cells were maintained in Dulbecco’s modified Eagle’s medium (10% heat-inactivated FBS). Antibiotics penicillin and streptomycin were added and were placed at 37 °C in a 5% CO_2_ incubator for colorimetric based assay using MTT (3-[4,5-dimethylthiazol-2-yl]-2,5-diphenyltetrazolium bromide) compounds **N9** and **N18** were seeded with five thousand HEK-293 cells (viability 98%) into 96-well plate for 24 h. Wells were added with MTT 5 mg/mL after 24 h incubation for 4 h [20]. Absorbance at 580 nm was recorded using Synergy/HTX MultiScan reader (BioTek) and lethal dose LD_50_ was calculated and for selectivity index (SI) was calculated.

## Structure–activity relationship studies

### Electronic discussion

The substitution pattern of the 2MBI benzamide derivatives was carefully selected to confer different electronic environment to the molecules. Thus, electron donating groups to aromatic ring, such as methyl and methoxy and electron withdrawing groups from aromatic ring, such as halogens, nitro were chosen as substituents on the molecular structure of the target compounds [20]. Most of 2MBI benzamides derivatives were found to possess moderate antimicrobial activity except those compounds which has electron withdrawing groups. Suggestions are made that the negative inductive effect plays a significant role in medicinal field. This means that compounds with high electron density gave poor antimicrobial activity which makes the diffusion of compounds more difficult throw the body of the bacteria cell while presence of electron withdrawing groups will cause a decrease of electronic density in 2MBI benzamides compared with electron donating groups, thereby facilitating entry of the 2MBI benzamides into the cell. This is likely to increase the antimicrobial potency. The increase in antimicrobial activity is due to faster diffusion of the 2MBI benzamides with electron withdrawing groups because the reducing the total electron density on 2MBI benzamide compounds make the diffusion faster through the bacteria cell [21].

**Fig. 6:**
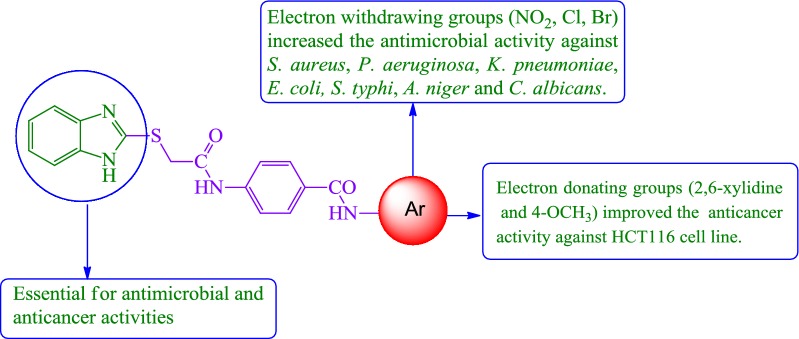
Structural requirements for the antimicrobial and anticancer activities of synthesized benzimidazole analogues

Because of their significant medicinal importance, the synthesis of substituted benzimidazoles has become a focus of synthetic organic chemistry. The antiproliferative effect and mechanism of induction of apoptosis by various bioactive heterocyclic compounds and the impact of electron releasing and withdrawing groups on the induction of apoptosis is supported by the studies of Gowda et al. [22]. Structure–activity relationship studies of the synthesized compounds based on electronic discussion, antimicrobial and anticancer screening results, the following SAR (Fig. 6) can be assumed:

i. Substitution of aromatic ring with 2,6-dimethyl substituent (compound **N9**, IC_50_ = 5.85 µM) considerably improved the anticancer activity of benzamide derivatives whereas substitution with 2,4-dimethyl group (compound **N10**, IC_50_ = 23.23 µM) had no such effect. The *para*-OCH_3_ substituted aromatic ring (compound **N18**, IC_50_ = 4.53 µM) significantly improved the anticancer activity of benzamide derivatives while the *ortho* and *meta*-OCH_3_ substituents (compound **N16–N17**, IC_50_ = 23.12 µM) does not improved the anticancer activity of synthesized compounds.
ii. The substitution of aromatic amine with di-NO_2_ group (compound **N1**) had significant effect on *B. subtilis*, *S. typhi* and fungal strain *C. albicans*, compared to compounds with single –NO_2_ substitution (**N2**, **N3** and **N4**). The *meta*-Cl and -Br substituted aromatic amines (compounds **N8**, **N22**) resulted in improved antimicrobial activity against *E. coli*, *K. pneumoniae* and fungus *A. niger*, respectively. The 2,3 and 2,5-dichloro substituted aromatic amines (compounds **N23**, **N25**) noticeably improved the antibacterial activity against *S. aureus*.

Thus, it very well may be stated that nitro, chloro and bromo substituents bearing derivatives are the most suitable scaffolds for accomplishing the best antimicrobial range. The role of electron withdrawing group in improving antimicrobial activities is supported by the studies of Kumar et al. [19].

## Experimental section

The reactants and reagents for the synthesis were got from Hi-media Laboratory, Loba Chemie and CDH Pvt. Ltd. Microbial type cell cultures (MTCC) for biological study were procured from Institute of Microbial Technology and Gene bank, Chandigarh. Reaction steps forward was monitored by thin layer chromatography (TLC) using ethyl acetate as mobile phase. The synthetic scheme was drawn via Chem Draw 8.03. Determination of melting point was done using labtech melting point equipment. IR spectrum was recorded on Bruker 12060280, spectrometer via KBr in the range of 4000–400 cm^−1^. ^1^H/^13^C-NMR spectra recorded at 600 and 150 MHz, respectively on Bruker Avance III 600 NMR spectrometer. For Mass spectra, Waters Micromass Q-ToF Micro instrument was used. Elemental analyses of 2MBI derivatives were performed on Perkin-Elmer 2400 C, H and N analyzer.

### Procedure to synthetic Scheme 1

#### Step i: Synthesis of 4-(2-chloroacetamido) benzoic acid (a)

*p*-Aminobenzoic acid (0.01 mol) and triethylamine (0.01 mol) were stirred properly in ethanol to get a clear solution and cooled it for half an hour. Now the solution was added with chloroacetylchloride (0.01 mol) and stirred for 1 h and the precipitated compound **a**, was strained via filtering, desiccated and recrystallized with ethanol [1].

#### Step ii: Synthesis of 4-(2-(1H-benzo[d]imidazol-2-ylthio)acetamido)benzoic acid (b)

Amixture of 4-(2-chloroacetamido) benzoic acid (**a**, 0.01 mol), 2-mercapto benzimidazole (0.01 mol) and potassium carbonate (0.01 mol) in ethanol (50 ml) was refluxed for 5–6 h and then cooled at room temperature followed by evaporation to dryness. The resultant residue was washed with water and recrystallized from ethanol [23].

#### Step iii: Synthesis of 4-(2-(1H-benzo[d]imidazol-2-ylthio)acetamido)benzoyl chloride (c)

Thionyl chloride (0.3 mol) was added to 4-(2-(1*H*-benzo[*d*]imidazol-2-ylthio) acetamido) benzoic acid (**b**, 0.25 mol) and the mixture were refluxed for 2–3 h . The excess of thionyl chloride was removed by distillation [17].

#### Step iv: Synthesis of final (N1-N26) 2MBI derivatives

The reaction mixture of 4-(2-(1*H*-benzo[*d*]imidazol-2-ylthio)acetamido)benzoyl chloride (**c**, 0.01 mol) and substituted aniline (0.01 mol) in suitable solvent was refluxed for appropriate time and thin layer chromatography was used to monitor the reaction. After completion of reaction, it was poured into ice cold water and the resultant precipitate was filtered, desiccated and recrystallized using ethanol [24].

## Biological study

### Antimicrobial evaluation (in vitro)

The antimicrobial potential of new synthesized compounds **(N1–N26)** was validated against Gram-positive, Gram-negative bacteria using cefadroxil and fungal strains with fluconazole, by serial dilution method. Dilutions were set up in nutrient broth I.P. for bacterial (incubated at 37 ± 1 °C for 24 h) and Sabouraud dextrose broth I.P. for fungal species (25 ± 1 °C for 7 days for *A. niger*) and (37 ± 1 °C for 48 h for *C. albicans*) [25, 26].

### Anticancer evaluation (in vitro)

The antiproliferative activity (expressed as IC_50_) was assessed against HCT116 using SRB B assay. This assay was based on the ability of SRB dye to bind electrostatically and its pH-dependence on protein basic amino acid residues of trichloroacetic acid-fixed cells [27]. Briefly, HCT116 was seeded at 2500 cell/well (96 well plates) and allowed to attach overnight before being exposed to 2MBI compounds (stock solution was suspended in DMSO) for 72 h and subjected to SRB assay. DMSO less than 1% did not kill the cells and the concentration of DMSO in each compound was 0.1%. Treated cells were fixed with 10% cold trichloroacetic acid and stained in 0.4% SRB. Unincorporated dye was rinsed off with 1% acetic acid and plates were left to air-dry at room temperature overnight. The air-dried plates were placed on a plate shaker and bound SRB was solubilised in 10 mM Tris base solution. Absorbance was measured by a computer-interfaced 96-well plate spectrophotometer at 570 nm.

### MTT assay

Human embryonic kidney (HEK-293) cells were maintained in Dulbecco’s modified Eagle’s medium (10% heat-inactivated FBS). Antibiotics penicillin and streptomycin were added and were placed at 37 °C in a 5% CO_2_ incubator for colorimetric based assay using MTT (3-[4,5-dimethylthiazol-2-yl]-2,5-diphenyltetrazolium bromide) compounds **N9** and **N18** were seeded with five thousand HEK-293 cells (viability 98%) into 96-well plate for 24 h. Wells were added with MTT 5 mg/mL after 24 h incubation for 4 h [28]. Absorbance at 580 nm was recorded using Synergy/HTX MultiScan reader (BioTek) and lethal dose LD_50_ was calculated and for selectivity index (SI) was calculated.

## Conclusion

**Fig. 7:**
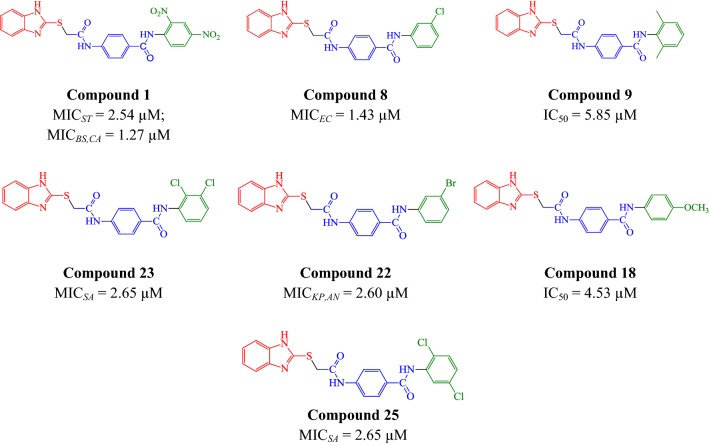
Molecular structures of the most active compounds

The present work involves the synthesis of new substituted benzamides linked to *p*-amino benzoic acid and 2-mercaptobenzimidazole which possess antibacterial, antifungal and antiproliferative activities. The synthesized compounds were evaluated in vitro against seven representative microorganisms along with the anticancer activity against carcinoma cell line (HCT116). Antimicrobial results demonstrated that the presence of nitro and halo groups in aromatic ring enhanced the antimicrobial potential. Anticancer results revealed that 4-(2-(1*H*-benzo[*d*]imidazol-2-ylthio)acetamido)-*N*-(2,6-dimethyl phenyl)benzamide (**N9**, IC_50_ = 5.85 µM) and 4-(2-(1*H*-benzo[*d*]imidazol-2-ylthio)acetamido)-*N*-(4methoxyphenyl) benzamide (**N18**, IC_50_ = 4.53 µM) were the most potent anticancer agents even higher than the standard drug 5-FU (IC_50_ = 9.99 µM). These compounds were more selective towards cancer cells rather than macrophages. Further the toxicity study revealed the better selectivity index against the HEK-293 cell lines at the respective IC_50_ concentration. Study suggested that compound may be safer as anticancer after required experimental evaluation. The molecular structures of active compounds are mentioned in Fig. 7.
